# A Synthesis of Viral Contribution to Marine Nitrogen Cycling

**DOI:** 10.3389/fmicb.2022.834581

**Published:** 2022-04-25

**Authors:** Shuai Wang, Yu Yang, Jiaojiao Jing

**Affiliations:** ^1^Shandong Collaborative Innovation Center for Diagnosis, Treatment and Behavioral Interventions of Mental Disorders, Institute of Mental Health, Jining Medical University, Jining, China; ^2^Key Laboratory of Shaanxi Province for Craniofacial Precision Medicine Research, College of Stomatology, Xi’an Jiaotong University, Xi’an, China; ^3^Department of Pediatric Dentistry, College of Stomatology, Xi’an Jiaotong University, Xi’an, China; ^4^Stomatological Center, Peking University Shenzhen Hospital, Shenzhen, China

**Keywords:** nitrogen cycle, marine viruses, microbial mortality, auxiliary metabolic genes, viral genomics

## Abstract

Nitrogen is an essential component of major cellular macromolecules, such as DNA and proteins. Its bioavailability has a fundamental influence on the primary production of both terrestrial and oceanic ecosystems. Diverse marine microbes consume nitrogen, while only a limited taxon could replenish it, leaving nitrogen one of the most deficient nutrients in the ocean. A variety of microbes are involved in complex biogeochemical transformations of nitrogen compounds, and their ecological functions might be regulated by viruses in different manners. First and foremost, viruses drive marine nitrogen flow *via* host cell lysis, releasing abundant organic nitrogen into the surrounding environment. Secondly, viruses can also participate in the marine nitrogen cycle by expressing auxiliary metabolic genes (AMGs) to modulate host nitrogen metabolic pathways, such as nitrification, denitrification, anammox, and nitrogen transmembrane transport. Additionally, viruses also serve as a considerable reservoir of nitrogen element. The efficient turnover of viruses fundamentally promotes nitrogen flow in the oceans. In this review, we summarize viral contributions in the marine nitrogen cycling in different aspects and discuss challenges and issues based on recent discoveries of novel viruses involved in different processes of nitrogen biotransformation.

## Introduction

Nitrogen cycle is an integral feature of marine nutrient cycles. As one of the most abundant elements in organic compounds, nitrogen affects the biosynthesis of pivotal cellular components, the activities of cellular metabolism, and the functioning of diverse ecosystems (Zehr and Kudela, 2011; Ward and Jensen, 2014). Nitrogen undergoes complex biogeochemical transformations in a cycle, which facilitate its bioavailability for a large variety of microbes in marine environments. Marine nitrogen cycle maintains the nitrogen homeostasis and connects with biogeochemical cycles of other elements, such as carbon, oxygen, and phosphorus (Gruber, 2008; Kuypers et al., 2018).

Nitrogen availability has a strong influence on the photosynthetic capacity, thus was considered as one of the major factors regulating marine primary production (Evans, 1989; Herbert, 1999). Nitrogen is not uniformly distributed across different biogeographic provinces or water columns in the ocean. Patterns of nitrogen vary from coastal estuaries to open ocean, from sunlit euphotic zone to the dark ocean (Saino and Hattori, 1987). In general, nitrogen is relatively low in the surface water, limiting primary productivity in the vast expanse of the pelagic ocean (Tyrrell, 1999). In contrast, estuary and coastal waters usually have higher primary productivity due to anthropogenic nitrogen inputs (Herbert, 1999).

**Figure 1:**
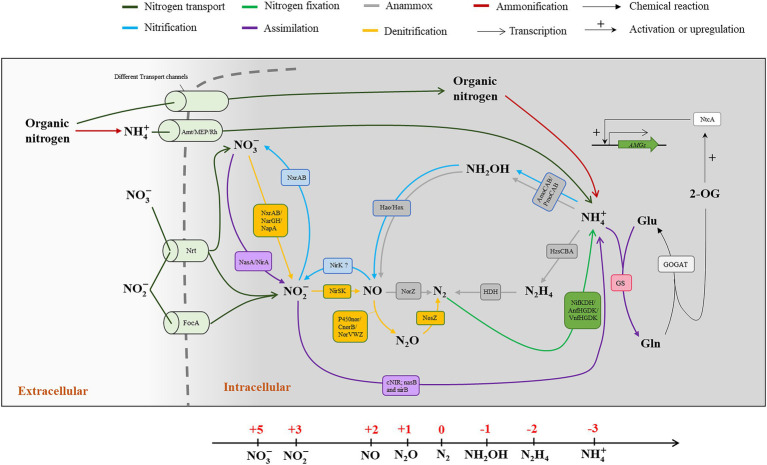
Biogeochemical transformations of nitrogen compounds in marine microorganisms. The nitrogen cycle consists of the transformations, including nitrogen transport nitrogen fixation, nitrification, denitrification, assimilation, ammonification, and anammox. The relevant cell localizations of these pathways are divided by a cytomembrane (Gray dotted line). These pathways are found in different microorganisms rather than limited to one single cell. The main pathways in nitrogen cycle and the participating enzymes and transporters are indicated in different colors. The oxidation valences of nitrogen atom, ranging from −3 to +5, in different compounds are indicated in a linear axis.

The nitrogen atom owns five valence electrons which can be flexibly arranged on electron orbitals in several stable oxidation states, ranging from −3 to +5 (Figure 1). In the marine environment, nitrogen could be commonly found in several chemical forms, such as ammonium ( ${\mathrm{NH}}_{4}^{+}$ , −3), hydrazine (N_2_H_4_, −2), hydroxylamine (NH_2_OH, −1), dinitrogen gas (N_2_, 0), nitrous oxide (N_2_O, +1), nitric oxide (NO, +2), nitrite ( ${\mathrm{NO}}_{2}^{-}$ , +3), and nitrate ( ${\mathrm{NO}}_{3}^{-}$ , +5; Figure 1). Microbes evolve diverse mechanisms for nitrogen uptake and transformation, including nitrogen fixation, nitrification, denitrification, anammox, and ammonification, and others (Zehr and Kudela, 2011; Kuypers et al., 2018; Hutchins and Capone, 2022; Figure 1), which are mainly happening in tiny microbial cells but have tremendous influence on the marine nitrogen budget. Despite dinitrogen gas being the most abundant nitrogen species on earth, it is only available to relatively a limited but diverse set of microbes that can fix N_2_ into biologically available ammonium. Ammonium, in which the nitrogen atom is fully reduced, is also the breakdown product during the decomposition or ammonification of organic matter. It is estimated that there is about 340–3,600 Tg nitrogen stockpiled in the form of ammonium in the ocean (Canfield et al., 2005; Gruber, 2008). Ammonium can also be transformed into other bioavailable oxidized nitrogen species, such as nitrite and nitrate through nitrification. Nitrate is also a crucial bioavailable form of nitrogen, which is particularly abundant in coastal waters where riverine inputs are significant, or where deep water is frequently brought to the surface layer by vertical advection processes. Acting as either electron donor or acceptor, nitrite could also be oxidized to nitrate by marine nitrifiers (Hutchins and Fu, 2017; Shafiee et al., 2019) or reduced to gaseous forms of nitrogen (N_2_O and N_2_) by denitrifiers (Kartal et al., 2007; Otte et al., 2019). N_2_O is another common form of nitrogen that is mostly produced by denitrifying (Zumft and Kroneck, 2007; Prosser et al., 2020) and ammonia oxidizing microbes (Shen et al., 2012; Jung et al., 2014). In addition to its ozone-depleting role, N_2_O is also a notorious greenhouse gas with a higher capability of trapping atmospheric heat than CO_2_, and its atmospheric concentration has been increasing mainly owing to anthropogenic emissions (Tian et al., 2020). In addition, there are a variety of organic compounds containing nitrogen in the forms of amino and amide groups in the oxidation state of −3. For example, urea and amino acids exist in the marine environment at low concentrations but can be rapidly recycled since they could be easily assimilated by both autotrophic and heterotrophic organisms (Baker et al., 2009).

Marine microbes constitute more than 90% of the living biomass in the ocean and are responsible for half of the global primary productivity (Field et al., 1998; Caron, 2005; Sogin et al., 2006; Bar-On et al., 2018). Viruses, the most abundant biological entities in the ocean, have also drawn extensive attention due to their roles in biogeochemical cycles since the discovery of significant viral contribution to microbial community turnover (Proctor and Fuhrman, 1990; Suttle, 1994). Lysogenic viruses can modulate microbial biodiversity and genome evolution through genetic exchange within and between hosts, while lytic viruses exert remarkable influence on microbial community structure and marine food web (Fuhrman, 1999; Wilhelm and Suttle, 1999) by turning infected host cells into dissolved and particulate organic matter (POM), which can then be utilized by other microbes (Middelboe et al., 1996; Middelboe and Lyck, 2002; Middelboe and Jorgensen, 2006). Although viral ecology is still in its infancy stage and quantification of the viral contribution to host mortality remains coarse and vague in natural environments, marine viral biodiversity has received considerable attention in recent years. There is a growing consensus that viruses act as crucial players in marine nutrient cycling and are indispensable for the maintenance of stable natural microbial communities and the functioning of ecosystems (Suttle, 2007; Jurgensen et al., 2021). Here, we review viral contribution to marine nitrogen cycling and present potential challenges and issues based on recent discoveries of novel viruses involved in different processes of nitrogen biotransformation.

## Viral Shunt Promotes Marine Nitrogen Flow Along With Microbial Loop

### The Interconnection of Viral Shunt With Microbial Loop in Marine Environment

About 40 years ago, the concept of “Microbial Loop (ML)” was introduced into the field of biological oceanography by Azam et al. (1983), pointing out the crucial role of marine microbes in bridging the gap between Dissolved Organic Matter (DOM) and classic marine food chain. DOM released by phytoplankton exudation, viral cell lysis, and zooplankton sloppy feeding was not directly available for marine macroplankton, but can be reutilized by marine microbes for proliferation. Microbes were further predated by larger marine organisms at higher trophic levels (such as protists) for growth; thus, nutrients can be recycled and transferred in the marine food chain with the help of microbes (Calbet, 2008; Fenchel, 2008).

**Figure 2:**
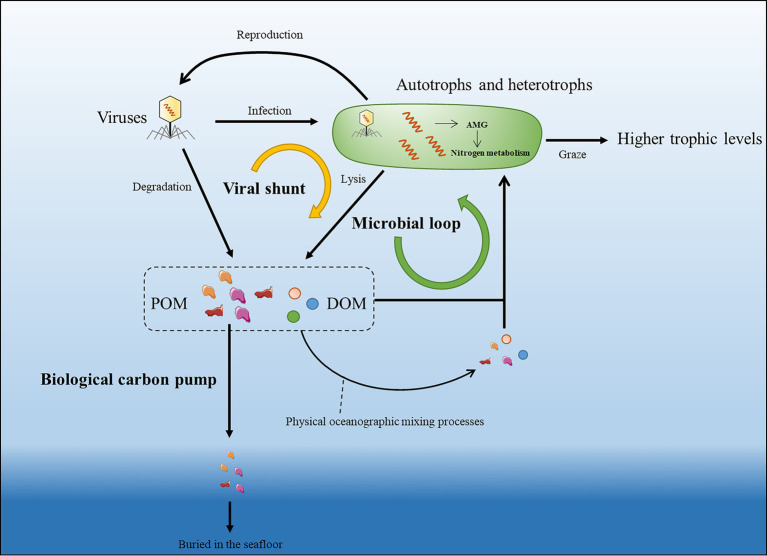
Schematic of viral participation in the marine nitrogen cycle. Virus-mediated lysis of microbial cells (viral shunt) and the degradation of viruses can release particulate organic matter (POM) and Dissolved Organic Matter (DOM) into the microbial loop. POM and DOM can be brought to different water columns through vertical ocean circulation, waves, and eddies pumping while a part of POM can also be sedimented to deep oceans by biological carbon pump. Upon infection, viruses can also regulate hosts’ nitrogen metabolism by encoding relevant auxiliary metabolic genes (AMGs).

Viruses were recognized as a new functional group responsible for material transfer and energy flow in the marine system (Proctor and Fuhrman, 1990; Bratbak et al., 1992; Thingstad et al., 1993). Virus-induced microbial mortality was termed as Viral Shunt (*VS*) by Wilhelm and Suttle (1999), describing the important role of viruses in breaking down microbial into DOM, which can be easily taken up by other microorganisms (Figure 2). Marine viruses lead to 20%–40% of microbial mortality in the surface ocean (Wommack and Colwell, 2000), and it was estimated that viral shunt contribute to over 25% of carbon recycling within the microbial loop in the global oceans (Wilhelm and Suttle, 1999).

Viruses are also important players of long-term marine carbon storage. Photosynthesis sequestrated carbon in the surface ocean support the marine food web, and a fraction of the surface fixed organic matter is eventually exported to the ocean interior and seafloor sediments by Biological Pump (BP; Figure 2). This vertical downward transfer mainly occurs through migrations of zooplankton and gravitational settling of microorganisms and POM (Boyd et al., 2019). Besides, a substantial fraction of organic matter does not reach the deep sea, but is remineralized in the water column by heterotrophic microbes, resulting in depletion of labile carbon and accumulation of recalcitrant carbon. This process was proposed as microbial carbon pump (MCP; Jiao and Zheng, 2011). Viruses are important participants in these “pumping processes.” First, viral infection can affect the sinking rates of microbial cells (Janice and Curtis, 2004) *via* enrichment of infected cells in sinking aggregates (Vincent et al., 2021). Moreover, virus can also influence the MCP-induced carbon sequestration by promoting microbial mortality (Vincent et al., 2021). Marine nitrogen flow is also affected by biological or microbial pumping processes. Particulate organic nitrogen (PON) can also sink into the deep ocean or can be converted back into ammonium by remineralization and subsequently used by microbes through nitrification (Jetten, 2008). The recycling of carbon and nitrogen is interactional as the fluctuating carbon-to-nitrogen ratios in the environment have a considerable impact on the microbial metabolism (Goldman et al., 1987; Shelford et al., 2012). Considering viral shunt is continuously changing the carbon/nitrogen ratio in the marine environment, viral contribution to nitrogen recycling seems to be more complicated to quantitatively estimate.

### Viruses Promote Nitrogen Recycling by Host-Specific Cell Lysis

Viruses exhibit multifaceted effects on nitrogen flow by controlling the biomass and bioactivity of diverse microbes involved in different nitrogen biogeochemical processes. Generally, viruses accelerate ammonification and assimilation by providing organic matter from lysates. Ammonification, the degradation process of organic nitrogen releasing ammonium, is widely found in bacteria, fungi, and algae, and its rate is mainly controlled by the abundance and composition of microbial communities, and by the availability of dissolved organic nitrogen. Dissolved low molecular weight organic compounds including amino acids, urea, amino sugars, and nucleotides are substrates for ammonification. These substrates can be first released from the depolymerization of macromolecules and are further transferred into bioavailable nitrogen by ammonification. On the contrary, assimilation can be briefly interpreted as the multi-step transformation of nitrogen from simple inorganic forms ( ${\mathrm{NO}}_{3}^{-}$ , ${\mathrm{NO}}_{2}^{-}$ , and ${\mathrm{NH}}_{4}^{+}$ ) into cellular biomass, such as amino acids and other organic compounds. The release of organic nutrients upon viral lysis may stimulate the growth of non-infected or unrelated microbes, which is crucial for the recycling of bio-limiting elements and structuring microbial communities. For example, viral lysates of *Vibrio* caused an increase in metabolic activity and cell production of the non-infected bacterioplankton, particularly in phosphorus-limited conditions (Middelboe et al., 1996). Isotope mass spectrometry analysis showed that viral lysis of *Phaeocystis globosa* enhanced ^13^C and ^15^N assimilation of *Alteromonas* by about 2.5-fold, and viruses enhanced bacterial substrate assimilation and indirectly shaped North Sea bacterial diversity (Sheik et al., 2014). Similarly, the degradation of *Phaeocystis pouchetii* lysates caused by virus PpV was reported to be associated with significant regeneration of inorganic N, which had strong positive effects on the abundance of heterotrophic bacteria and nanoflagellates, suggesting an efficient transfer of organic material from *P. pouchetii* to other microbes through viral lysis (Haaber and Middelboe, 2009).

Increasing evidence support the crucial functions of viruses in regulating nitrogen fixation by controlling the turnover of diazotrophic cyanobacteria. The significance of N_2_ fixation has been historically underestimated according to biogeochemical analysis (Michaels et al., 1996; Gruber and Sarmiento, 1997) even after the discovery of widespread diazotrophs, such as tropical/subtropical cyanobacterium *Trichodesmium* spp. (Capone et al., 1997) and symbiotic cyanobacterial species of some diatoms (Villareal, 1994) in the ocean. Convergent estimates indicate that microbial fixation of N_2_ delivers approximately 163 Tg N year^−1^ globally, which is far more than atmospheric deposition and riverine input (Wang et al., 2019). The amount of nitrogen fixed by *Trichodesmium* per year was estimated to be about 60–80 Tg N year^−1^ globally (Capone et al., 1997; Mahaffey et al., 2005; Westberry and Siegel, 2006), making up more than ⅓ of the annually fixed N_2_ in the global ocean (~100–200 Tg N year^−1^ according to Karl et al., 2002). However, the past estimates were based on the assumption that all species in the *Trichodesmium* genus were capable of fixing N_2_, which was overthrown by a very recent metagenomic study showing abundant yet non-diazotrophic *Trichodesmium* species are widespread in the open ocean (Delmont, 2021). This decoupling of functional traits from taxonomic lineages alerts further endeavors are needed to decipher *Trichodesmium* community composition, which likely affect the estimate of their contribution to global nitrogen balance. Viral infection of diazotrophic cyanobacteria, such as *Trichodesmium*, could result in large perturbations of dissolved and particulate organic matter in the surface ocean, leading to a shift in nitrogen availability for co-occurring microbes (Rodier and Le Borgne, 2010). Viral lysis is a crucial cause of the rapid collapse of *Trichodesmium* aggregations (Hewson et al., 2004). The appearance of virus-like particles in mitomycin C-treated *Trichodesmium* cells revealed that *Trichodesmium* harbor viruses that could enter the lytic cycle under some specific but as-yet undefined conditions (Ohki, 1999; Hewson et al., 2004). Collapse of *Trichodesmium* aggregations can lead to large releases of particulate and DOM from dying cells for co-occurring microbes in the environment (Rodier and Le Borgne, 2010). The bloom-forming diazotrophic filamentous cyanobacteria *Aphanizomenon flos-aquae* are often devoid of zooplankton grazing due to its toxin production (Rollwagen-Bollens et al., 2013). Viral based top down control may play a significant role in suppressing *Aphanizomenon flos-aquae* especially in eutrophic ecosystems (Simis et al., 2005). Previous studies found two cyanophages, Vb-AphaSCL131 and Vb-AphaM-CL132, belonging to *Siphoviridae* and *Myoviridae*, respectively, in the purified lysates of *Aphanizomenon flos-aquae* (Gelzinis et al., 2015; Sulcius et al., 2019). After cell lysis by Vb-AphaSCL131, substantial release of ammonium into the culture medium was detected (Kuznecova et al., 2020). Although the growth of *Aphanizomenon flos-aquae* was significantly inhibited by Vb-AphaSCL131 infection, *nifH* gene expression level and the nitrogen fixation rates are barely altered, as cyanophage replication and progeny production within vegetative cells does not interfere with the N_2_ fixation process in heterocysts. Intriguingly, Vb-AphaSCL131 can also reduce the transport of fixed nitrogen from heterocysts to vegetative cells, which leads to nitrogen accumulation at the poles of heterocysts (Kuznecova et al., 2020).

Viral lysis of nitrifying bacterium and ammonia oxidizing archaea (AOA) can also affect ammonia metabolism. Nitrification is the primary process of producing oxidized nitrogen species, such as ${\mathrm{NO}}_{3}^{-}$ , ${\mathrm{NO}}_{2}^{-},$ and N_2_O, from reduced nitrogen (NH_3_ or ${\mathrm{NH}}_{4}^{+}$ ). Nitrification widely occurs in the surface, subsurface, deep layer, and sediments in the ocean (Camila Fernandez and Patrick, 2007; Yool et al., 2007; Hutchins and Capone, 2022). The nitrification process is mainly performed by different microbes, including ammonia oxidizing bacteria (AOB), AOA, and nitrite-oxidizing bacteria (NOB; Ward, 2013). Besides, specific species of the bacterial genus *Nitrospira* are capable of performing both of the nitrification steps, called complete ammonia oxidation (Comammox; Daims et al., 2015; Van Kessel et al., 2015; Molina et al., 2018). The most common AOB in natural environments belong to the *Nitrosomonas*, *Nitrosospira*, and *Nitrosococcus* genera (Purkhold et al., 2000). AOA belong to the class Nitrososphaeria within the phylum *Thaumarchaeota*, which could be known as Nitrososphaerales, Nitrosopumilales, *Ca*. Nitrosotaleales, and thermophilic *Ca*. Nitrospina spp. (Stieglmeier et al., 2014). AOA from the phylum Thaumarchaeota play a prominent role in ammonia metabolism. Viral infection is suggested to represent a key mechanism controlling the turnover of archaea, especially AOA, in deep-sea sediments (Danovaro et al., 2016). Three *Nitrosopumilus* spindle-shaped viruses are isolated from suspended particulate matter-rich seawater samples and characterized to efficiently infect autochthonous *Nitrosopumilus* strains, leading to inhibition of AOA growth, accompanied by severe reduction in the rate of ammonia oxidation and nitrite reduction (Kim et al., 2019). Similarly, it was reported that the infection of a specific phage CM-1 induced rapid decline of the heterotrophic nitrifying bacterium *Lutimonas* sp. H10 and reduced the efficiency of ammonia removal in marine aquaculture (Fu et al., 2009).

## Viruses Influence Microbial Nitrogen Metabolism Through Encoding Auxiliary Metabolic Genes

Viruses can also modulate the marine nitrogen cycle by expressing auxiliary metabolic genes (AMGs) involved in host nitrogen metabolisms. Viruses encoded AMGs have been broadly discovered in different environments and are suggested to be involved in several different biogeochemical processes, including nitrification, denitrification, anammox, ammonia assimilation, and nitrogen transport (Supplementary Table S1).

### Viral-Encoded Enzymes Could Promote Marine Nitrification Process

Some viruses carrying AMGs can also modulate the nitrification pathway during infection. Genes like *amo*A/B/C, encoding for ammonia monooxygenase enzyme, catalyzing the first step of ammonia oxidation and have been regarded as biomarkers for nitrifiers in the oceans (Junier et al., 2010). Ahlgren et al. (2019) discovered 15 new genomically and ecologically distinct viral populations infecting marine Thaumarchaeota (potentially tailed viruses that share a common ancestor with related marine Euryarchaeota viruses). These viruses bear thaumarchaeal ammonia monooxygenase genes (*amoC*) in globally various marine habitats. Metagenomics provided evidence that viral *amoC* sometimes comprise up to half of total *amoC* DNA copies, indicating their potential roles in nitrification processes (Roux et al., 2016; Ahlgren et al., 2019). Similarly, two archaeal-like *amoC* genes and one bacterial-like *amoC* were also identified in viruses isolated in Eastern Tropical South Pacific oxygen minimum zone (ETSP OMZ) waters while the functional roles remain to be resolved (Gazitua et al., 2021).

### Viral-Encoded AMGs in Denitrification Pathways

Canonical denitrification refers to several respiratory reactions that use organic nitrogen compounds as electron acceptors for the production of nitrite, nitric oxide, nitrous oxide, and dinitrogen gas, finally leading to the loss of N from the marine system. Denitrification occurs in specific habitats, such as the oxic–anoxic interface of benthic sediments, and suboxic or anoxic waters where nitrate is present but oxygen is deficient (Naqvi et al., 2008). Nitrate is generally the first electron acceptor in the process of denitrification especially in hypoxic column while organic nitrogen can also be reduced *via* heterotrophic denitrification. Microbes carry out different denitrification processes based on the availability of nitrogen resources and the redox gradients present in the environment (Tesoriero et al., 2021). Periplasmic nitrate reductase (Nap), respiratory nitrate reductase (Nar), and assimilatory nitrate reductase (Nas) catalyze the reaction of dissimilatory nitrate reduction to nitrite. Subsequently, nitrite can be reduced into nitric oxide by nitrite reductase (Nir). Nitric oxide (Nor) reductase induces the reduction of nitric oxide to nitrous oxide or dinitrogen gas. Nitrous oxide can be an intermediate or end product during denitrification (Suntharalingam et al., 2000). Metagenomic analysis revealed a ferredoxin-nitrite reductase gene *nirA*, a copper-containing nitrite reductase gene *nirK*, and a nitric oxide reductase gene *norB* as viral-encoded AMGs in ETSP OMZ waters. For example, a *nirA* gene that is similar to the homolog in *Prochlorococcus* and *Synechococcus* exits in the genome of a potential T4-like cyanomyophage (Gazitua et al., 2021). Viral NirA may attenuate their host’s need to compete for limited ammonia in some particular environments, such as Peruvian anoxic marine zone (AMZ; Lam et al., 2009) and OMZ over the Omani Shelf (Jensen et al., 2011; Ulloa et al., 2012). Besides, contiguous *nirK* and *norB* are also identified in a unit within the genome of a myovirus according to the taxonomic annotation of the viral-like genes. Motif analysis and structural prediction indicate the functional activity of viral NirK and NorB revealing their participation in hosts’ denitrification processes during infection (Gazitua et al., 2021).

### Viruses May Compensate Ammonium Oxidation of Anaerobic Hosts

Along with denitrification, the anaerobic oxidation of ammonium (anammox) is another anaerobic process that removes bioavailable nitrogen from the water. Anammox bacteria obtain energy and electrons from ammonia oxidation anaerobically. In an anammox reaction, ammonium and nitrite are combined to form N_2_, generating NO and hydrazine (N_2_H_4_) as two key intermediates (Kartal and Keltjens, 2016; Soler-Jofra et al., 2021). In the first step, the one-electron reduction of nitrite to NO is catalyzed by nitrite reductases (NIR) which exist in many denitrifying microorganisms. The second reaction combines ammonium and NO to produce hydrazine which is catalyzed by hydrazine synthase (HZS) (Strous et al., 2006). Finally, hydrazine is converted into N_2_ by hydrazine oxidoreductase (HZO; Dalsgaard et al., 2014). So far based on isolation and metagenomic surveys, 19 species under six candidate genera, including *Brocadia*, *Scalindua*, and *Kuenenia*, have been reported in various natural and synthetic ecosystems, such as marine sediments, hydrothermal vents, sponges, and anoxic waters (Oshiki et al., 2016; Zhang and Okabe, 2020). *Nir* genes were also detected as viral AMGs in the anoxic water column, indicating their functional roles in modulating anammox reaction by promoting NO production (Gazitua et al., 2021). Viruses are believed to have compensation effects on the nitrogen metabolisms of their host cells. Viral *nar* genes are also identified in the samples from deep-sea hydrothermal vent sediment in the Southwest Indian Ocean. As revealed by the metabolic pathways in viromes and microbiomes studies, histidine kinase NarX, a nitrate–nitrite sensor, sensed the extracellular level of nitrate or nitrite and then activated the response regulator NarL, which leads to the gene expression inhibition of microbial nitrite reductase, including NarG, NarH, NarJ, and NarI subunits, in microbial nitrogen metabolism (He et al., 2017). Metabolic compensation of hosts mediated by viruses may help hosts to adapt to extreme environments and may be essential for host survival.

### Viral-Encoded AMGs Influence Host Ammonia Assimilation

Ammonia assimilation is the process of incorporating ammonia into organic matter. The conversion of ammonium into amino acids (glutamate and glutamine) is initially catalyzed by glutamate synthetase and glutamine synthetase, respectively. Glutamate synthetase catalyzes the reaction of glutamate production from glutamine and 2-oxoglutarate (2-OG), and glutamine synthetase is responsible for the formation of glutamine by catalyzing the condensation of glutamate and ammonium. Viromes from the surface, oxycline, and anoxic zones in ETSP identified several viral AMGs, such as glutamate synthase and glutamine synthetase, revealing potential participation of these viruses in ammonia assimilation and nitrogen metabolism (Cassman et al., 2012). Sullivan reported that 16 marine cyanobacterial myoviruses isolated from a variety of locations across tropical and subtropical oceans contain at least one and often numerous hypothetical proteins with possible phytanoyl-CoA-dioxygenase domains, which may act on 2-OG as oxidoreductases to regulate ammonia assimilation, while 14 of the marine cyanophages contained numerous 2-OG-FeII oxygenase superfamily proteins. Besides, all 16 genomes contain numerous NtcA binding sites, which are involved in promoting a diversity of both T4 phage and cyanophage genes. These pieces of evidence suggest that these cyanobacterial myoviruses can modulate 2-OG levels to influence ammonia assimilation and stimulate NtcA activity which is needed to promote phage genes expression (Sullivan et al., 2010).

### Viruses Encode Diverse AMGs Involved in Nitrogen Transport

Microbes have evolved a range of strategies including effective nitrogen uptake and transport to optimize nutrient acquisition and adapt to environmental limitations. The transport of ammonium is mediated by the ammonium transporter/methylammonium permease/rhesus (Amt/Mep/Rh) family. Amt/Mep/Rh family is responsible for the bidirectional diffusion of ammonium across the membrane and is necessary for microbial growth at low ammonium concentrations. A host-derived ammonium transporter gene Amt was identified in the genome of a phytoplankton virus, which infects the small green alga *Ostreococcus Tauri*. The viral gene is demonstrated to be transcribed during infection and the viral protein can transport ammonium, methylammonium, and potentially a range of alternative N sources (Monier et al., 2017). Similarly, targeted metagenomics based on cell sorting revealed Amt/Mep/Rh family genes in the genomes of two viral strains of giant Mimiviridae sampled from the North Pacific Ocean (Needham et al., 2019). Besides, three viral populations harboring the *glnK* gene followed by the *amtB* gene were also identified in the ETSP OMZ (Gazitua et al., 2021). GlnK is a PII signal transduction protein linked to AmtB (ammonia channel protein). Under nitrogen-limiting conditions, GlnK is covalently modified by uridylylation to inhibit the interaction with AmtB and enable the uptake of ammonium (Gazitua et al., 2021). Moreover, nitrite transporter gene (*focA*) was also previously detected in the viral genome of a potential T4-like cyanomyophage. FocA, a representative nitrite transporter from the formate/nitrite family is universally found in bacteria, proteobacteria, archaea, fungi, algae, and parasites (Wang et al., 2009). Potential expression of this gene during infection may promote nitrite uptake of the hosts (Gazitua et al., 2021).

## Viruses Provide Considerable Reservoirs of Nitrogen Element

Viral particles are also a considerable reservoir of nitrogen element given their high abundance in the ocean. It is predicted that a bacterial cell contains at least two orders of magnitude more carbon than a virus particle (Bertilsson et al., 2003). However, the relative content of nitrogen in viruses is quite different compared with their hosts, since a virus particle is predominantly composed of a capsid (mostly protein) and core genetic materials (nucleic acids). The carbon/nitrogen/phosphorus (C/N/P) ratio was estimated to be 106/16/1 for marine plankton (Redfield et al., 1963; Jiao et al., 2010, 2011). This ratio vary greatly among different organisms, which is about 69/16/1 in marine heterotrophic bacteria (Suttle, 2007), about 46/10/1 in *Prochlorococcus* sp. MED4 and 301/49/1 in *Synechococcus* sp. WH8013 under phosphorus-limited conditions (Bertilsson et al., 2003). However, the C/N/P ratio in *Paramecium bursaria Chlorella* virus 1 (PBCV1) was estimated to be 17/5/1 (Clasen and James, 2007), highly enriched in nitrogen and phosphate in bacteriophages (Jover et al., 2014). Marine viruses are estimated to contain 0.055–0.2 fg carbon per virus particle in different studies (Wilhelm and Suttle, 1999; Suttle, 2005, 2007; Steward et al., 2007), and nitrogen content is estimated ranging from 0.0078 to 0.02 fg (Jover et al., 2014). The viral contribution to the DON pool could range up to 7% in the marine system if the virus densities can reach up to 10^11^ per liter.

The viability of viruses is tightly controlled in the marine environment. For example, solar radiation (especially UV-B irradiation) is considered as a causative factor responsible for defective viruses in the surface ocean (Suttle and Chen, 1992; Noble and Fuhrman, 1997). Full sunlight can lead to an average loss of viral infectivity of 20%/h for a diverse range of marine virus isolates (Mojica and Brussaard, 2014). Temperature and salinity are also responsible for differences in viral growth rate and delay of infection (Mojica and Brussaard, 2014). Moreover, extracellular enzymes produced by microorganisms can also degrade viral particles into dissolved organic nitrogen. Additionally, viral particles can also be absorbed onto particles and then vertically sink to the deep ocean or are transported laterally with the ocean currents (Wommack and Colwell, 2000; Weinbauer, 2004). These activities account for the exchange of free-living viral particles in different water columns, making viruses not restricted to the local biogeochemical cycling.

## Challenges and Prospects

The topic of viral implications on the cycling of organic matter in the marine ecosystem caught the early attention of researchers and has been widely studied globally for over half a century. However, the ecological roles of diverse marine viruses in the nitrogen cycling or other host metabolism are generally understudied, particularly in the context of virus–host interactions. Difficulties in sampling over the large temporal and spatial scales, and the dependence of cultured hosts for viral isolation and culture, especially from less accessible environments, such as hydrothermal vents or anoxic waters, hindered the study of their participation in the nitrogen cycle and other ecological functions, despite metagenomics and mathematical modeling have been proved to be helpful to identify and characterize viral diversity and to explore the mechanism of interactions between viruses and their hosts. Isolation and cultivation are necessary for quantifying viral and host physiological capabilities; however, beyond the limitation of host availability, it is also arguable that virus–host interactions in the laboratory might be different from the natural environment, where population size and abiotic factors might affect their interactions.

Some growth-limiting factors, such as temperature, oxygen, light, and nutrients, are proven to be crucial for host–virus interactions. It is still an exciting but challenging problem to identify the factors controlling the temporal and spatial influence on the nitrogen cycle of viruses (Maat et al., 2014). Nitrogen cycling is inevitably interconnected with many other elements, most notably, such as carbon, phosphorus, and sulfur. For example, increasing bioavailable nitrogen in the ocean can fertilize the ocean’s biosphere and enhance the uptake of CO_2_ by photosynthetic microorganisms and phytoplankton. However, ocean acidification resulting from the mounting uptake of anthropogenic CO_2_ can exert huge effects on a variety of marine life (Langdon et al., 2003; Kapsenberg and Cyronak, 2019; Melzner et al., 2020) and lead to an uncharted disturbance of C/N ratio which is important for balance of marine ecosystem. It was reported that ocean acidification could lead to enhanced photosynthesis (Riebesell et al., 2007) and nitrogen fixation by *Trichodesmium* (Barcelos E Ramos et al., 2007). According to a current meta-analysis incorporating 49 publications, diazotrophic nitrogen fixation is estimated to be enhanced by 29 ± 4% while nitrification processes are estimated to be reduced by 29 ± 10% under ocean acidification by 2,100 (Wannicke et al., 2018). However, the viral participation in the coupling cycle of C/N is rarely studied. Considering the complicated flow of nitrogen with other elements, a combination of observational, experimental, and modeling approaches could provide more comprehensive information for our understanding of the viral effect on marine nitrogen cycle. Moreover, sulfur cycling, primarily driven by microbial reduction of sulfate to produce hydrogen sulfide, is also regarded as an influential biochemical process globally. Sulfate reduction is one of the major processes in deep-sea columns especially in marine sediments (Bell et al., 2020). As a key agent in the sulfur cycle, viruses also alter sulfur metabolism within host cells during infection *via* expressing AMGs involved in relevant processes (Anantharaman et al., 2014; Coutinho et al., 2020; Flieder et al., 2020; Kieft et al., 2020, 2021). It can be speculated that the study of viral activities in coupled cycling of nitrogen and sulfur is another hot topic in the future.

The study of marine eukaryotic viruses is a less tapped field that deserves more attention. Single-celled microeukaryotes and small multicellular zooplankton accounting for a substantial fraction of the planktonic biomass in the ocean, and are discovered to be involved in various processes of biogeochemical cycles of major elements (Dortch and Packard, 1989; Gasol et al., 1997; Barton et al., 2013). Due to their large genomes, enormous diversity, and largely unexplored physiologies, the functional significance of marine microbial eukaryotes as well as viruses infecting them are difficult to investigate. A study of the samples from *Tara* Oceans using metatranscriptomic approach revealed an impressive number of genes without functionally characterized homologs in available databases (Carradec et al., 2018). At-sea flow cytometry with staining and sorting seems to be an effective method to isolate eukaryotic viruses (Needham et al., 2019), and a combination of metagenomics and metatranscriptomics approaches may provide more insight into the roles of marine eukaryotic viruses in the future.

Exploring the complete marine food web trophic continuum from viruses to top predators is ecologically important but not fully resolved, and the stable isotope method is a potentially useful method for further study (Cai et al., 2005). A promising new application of stable isotope technology is the visualization of isotope labeling at the cellular level through nanoscale secondary ion mass spectrometry (nanoSIMS; Finzi-Hart et al., 2009; Taylor, 2019). Multiple stable isotopes can be distinguished in high-resolution images by NanoSIMS to track nitrogen transfer between cells or cell structures (Foster et al., 2011; Thompson et al., 2012; Bonnet et al., 2016; Taylor, 2019).

The quantification of viral contribution to marine nitrogen cycling remains challenging due to the variable content of viral mass in different water column and dynamic interactions between viruses and the hosts. For example, marine viruses can affect the elemental assimilation rates of their infected hosts, while altered host post-infection metabolism can also influence the viral activities (Zeng and Chisholm, 2012). It is necessary to build different biophysical models to provide more insight into the potential dynamics in virus–host interactions. A biophysical scaling model based on sequence and structural information of different viruses is validated to quantify the elemental stoichiometry of marine viruses in the ocean (Jover et al., 2014). In summary, the conjunction of classic morphological (Lindell et al., 2005), biophysical, and biogeochemical (Clasen et al., 2008) techniques with deep metagenomics and metatranscriptomics analysis are considered to be the rigorous method to quantify the relative contribution of viruses to the pools and fluxes of marine energy and materials.

## Concluding Remarks

Viruses are the most abundant and diverse biological entities in the global ocean, yet their ecological roles and biogeochemical contributions in the marine environments are still not well understood due to limitations of sampling scales and research methods. Broadly, viruses fundamentally influence nitrogen recycling by inducing microbial mortality, and they also constitute a noteworthy part of marine nitrogen inventory. Intriguingly, viruses can also regulate biogeochemical nitrogen metabolisms in hosts *via* controlling AMGs expression. Future viral ecology requires more effort putting into large-scale and long-term sampling, new techniques for virus and host isolation, sorting, and culture, and more elaborate single viral genomics.

## Author Contributions

SW and JJ wrote the manuscript and made the figures. JJ, SW, and YY revised the manuscript. All authors contributed to the article and approved the submitted version.

## Funding

This study was supported by the Department of Science and Technology of Shaanxi Province (2019JQ-972), the Fundamental Research Funds for the Central Universities (xzy012019091) to JJ, the Research Fund for Academician Lin He New Medicine (JYHL2019MS01), the Research Fund from Key Laboratory Project of Zhejiang Province (ZJAD-2021001) to SW, and the Traditional Chinese Medical Project of Shandong Province (2021Q065) to YY.

## Conflict of Interest

The authors declare that the research was conducted in the absence of any commercial or financial relationships that could be construed as a potential conflict of interest.

## Publisher’s Note

All claims expressed in this article are solely those of the authors and do not necessarily represent those of their affiliated organizations, or those of the publisher, the editors and the reviewers. Any product that may be evaluated in this article, or claim that may be made by its manufacturer, is not guaranteed or endorsed by the publisher.

## Supplementary Material

The Supplementary Material for this article can be found online at: https://www.frontiersin.org/articles/10.3389/fmicb. 2022.834581/full#supplementary-material
